# Applications of Transistor-Based Biochemical Sensors

**DOI:** 10.3390/bios13040469

**Published:** 2023-04-11

**Authors:** Qiya Gao, Jie Fu, Shuang Li, Dong Ming

**Affiliations:** 1 Academy of Medical Engineering and Translational Medicine, Tianjin University, Tianjin 300072, China; 3019207445@tju.edu.cn (Q.G.); fujie314159@tju.edu.cn (J.F.); richardming@tju.edu.cn (D.M.); 2 Department of Biomedical Engineering, College of Precision Instruments and Optoelectronics Engineering, Tianjin University, Tianjin 300072, China

**Keywords:** biochemical sensor, field-effect transistor (FET), organic field-effect transistor (OFET), organic electrochemical transistor (OECT), intelligent point-of-care testing (iPOCT)

## Abstract

Transistor-based biochemical sensors feature easy integration with electronic circuits and non-invasive real-time detection. They have been widely used in intelligent wearable devices, electronic skins, and biological analyses and have shown broad application prospects in intelligent medical detection. Field-effect transistor (FET) sensors have high sensitivity, reasonable specificity, rapid response, and portability and provide unique signal amplification during biochemical detection. Organic field-effect transistor (OFET) sensors are lightweight, flexible, foldable, and biocompatible with wearable devices. Organic electrochemical transistor (OECT) sensors convert biological signals in body fluids into electrical signals for artificial intelligence analysis. In addition to biochemical markers in body fluids, electrophysiology indicators such as electrocardiogram (ECG) signals and body temperature can also cause changes in the current or voltage of transistor-based biochemical sensors. When modified with sensitive substances, sensors can detect specific analytes, improve sensitivity, broaden the detection range, and reduce the limit of detection (LoD). In this review, we introduce three kinds of transistor-based biochemical sensors: FET, OFET, and OECT. We also discuss the fabrication processes for transistor sources, drains, and gates. Furthermore, we demonstrated three sensor types for body fluid biomarkers, electrophysiology signals, and development trends. Transistor-based biochemical sensors exhibit excellent potential in multi-mode intelligent analysis and are good candidates for the next generation of intelligent point-of-care testing (iPOCT).

## 1. Introduction

Biochemical sensors are analytical devices that detect specific targets by converting the biochemical molecular recognition process into amplified and measurable physicochemical signals [1]. Biochemical sensors serve dual functions as receivers and converters. They are composed of immobilized sensitive materials as recognition elements, appropriate physicochemical transducers, and signal amplification devices. The recognition elements include enzymes, antibodies, antigens, micro-organisms, cells, nucleic acids, and metabolites [2,3,4,5,6,7]. The physicochemical transducers include electrodes, photoelectric converters, field-effect transistors, and piezoelectric crystals [8,9,10,11,12,13]. The recognition element of a biochemical sensor responds to target materials that generate a detectable electrical signal. This signal depends on the analyte concentration and biomedical solution properties. The advantages of using biochemical sensors to detect target biomolecules include rapid response, easy operation, accurate results, relatively low cost, and so on. These advantages and continuous innovation in biochemical sensor technology have attracted researchers’ interest in developing biochemical sensors for target analyte detection under various physiological conditions. Wearable and implantable sensors can monitor various human health indicators and offer insight into kidney, cardiovascular, and respiratory system diseases. These technologies help facilitate early prevention and proper treatment [14,15,16].

Point-of-care testing (POCT) is an emerging medical care field that analyzes and diagnoses in vitro with quick results. POCT does not require specialized personnel to perform and is not limited by the environment. It can be performed on the spot, at home, in ambulances, or in hospitals [17,18]. With the large-scale outbreak of infectious diseases such as COVID-19, POCT plays an essential role in the rapid diagnosis and timely screening of diseases, especially in areas with limited resources [19]. In this case, biochemical sensors are convenient for POCT due to their high selectivity and specificity. For these reasons, portable biochemical sensors for POCT can significantly improve medical detection efficiency.

In addition to their high sensitivity, rapid response, and reasonable specificity, transistor-based biochemical sensors have been used to detect different biochemical molecular targets because they are portable, simple to operate, and do not require the pretreatment of analytes. In recent years, transistor-based biochemical sensors have also been used to detect human physiological markers, such as ECG signals, body temperature, etc., due to their superior signal amplification capability at low voltage and power consumption. As highly sensitive sensor devices, transistor-based biochemical sensors have great potential when combined with artificial intelligence (AI). Using machine learning (ML) methods to design devices and quantitatively analyze biological signals measured by transistor-based biochemical sensors may greatly improve detection accuracy and efficiency [20,21,22,23,24]. These developments will help transistor-based biochemical sensors pave the way for the next generation of point-of-care testing [25].

With the emergence of organic materials and nano-materials, transistor-based biochemical sensors have continuously improved with more advantages in detecting target molecules. In this paper, we introduce three kinds of transistor-based biochemical sensors and their applications, namely the field-effect transistor (FET), organic field-effect transistor (OFET), and organic electrochemical transistor (OECT). These biosensors have distinct advantages when detecting biomolecules in body fluids (such as cortisol, glucose, dopamine, lactate, K^+^, Ca^2+^, and H^+^) [11,26,27,28,29] and electrophysiology (ECG signals, body temperature) [30,31,32]. To clarify the concept of transistor-based biochemical sensors, we also briefly introduce the fabrication process of sensor electrodes.

## 2. Principles of Transistor-Based Biosensors

**Figure 1:**
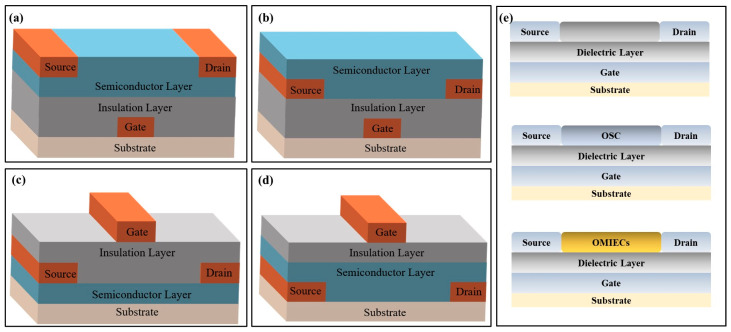
Structural diagrams of transistor-based biosensors. (**a**) Bottom-gate/bottom-contact transistor-based sensor. (**b**) Bottom-gate/top-contact transistor-based sensor. (**c**) Top-gate/bottom-contact transistor-based sensor. (**d**) Top-gate/top-contact transistor-based sensor. (**e**) Schematics of three transistor-based biochemical sensors.

Biochemical sensors based on transistors consist of two parts: (1) signal transduction and amplification elements and (2) signal recognition elements, which convert biochemical signals into measurable and observable electrical signals. The transistor-based biochemical sensors structure contains a substrate, insulation layer, semiconductor layer, gate electrode, and source/drain (S/D) electrode. Gate electrodes can be divided into two types based on their position: top electrode and bottom electrode structures. They include bottom-gate/bottom-contact, bottom-gate/top-contact, top-gate/bottom-contact, and top-gate/top-contact (Figure 1a–d). Bottom-gate refers to the gate deposited below the insulation layer, and the top-gate refers to the gate deposited above the semiconductor and insulation layers. Top-contact and bottom-contact are divided according to the semiconductor and source/drain electrode positions. In top-contact, the semiconductor grows on the insulation layer where the S/D electrodes are deposited. In contrast, bottom-contact refers to S/D electrodes above the semiconductor layers. When target molecules are detected by transistor-based biochemical sensors, the gate’s voltage changes and is conducted through the bottom- or top-gate.

In a transistor-based biochemical sensor, the gate metal film can be replaced by a biochemical-sensitive film. When analytes act on the transistor component or interface, the biochemical-sensitive film’s composition, stacking mode, or charge density will change. This reaction affects the sensor’s electrical signal output and ultimately achieves higher detection sensitivity. Transistor-based biochemical sensors can be divided into FET, OFET, and OECT biochemical sensors based on their modified materials and specific structures. FET’s versatility arises from inorganic semiconductor materials (metal oxides and ion-selective membranes) or biomolecules directly related to the species being examined. OFET’s versatility is derived from organic semiconductor materials (OSC). While OECT’s versatility mainly depends on the material synthesis and functionalization of organic mixed ionic/electronic conductors (OMIECs) (Figure 1e).

## 3. Electrodes of Transistor-Based Biosensors

Electrodes are important components of transistor-based biochemical sensors. Conductivity, resolution, thinness, and smoothness are the key factors that determine whether sensors detect targets accurately. Therefore, the fabrication and processing technology of electrodes has become a critical step in producing transistor-based biochemical sensors. Common transistor electrode manufacturing processes include inkjet printing, screen printing, laser ablation, and lithography [33,34,35,36,37]. Some examples of these methods are described below.

**Figure 2:**
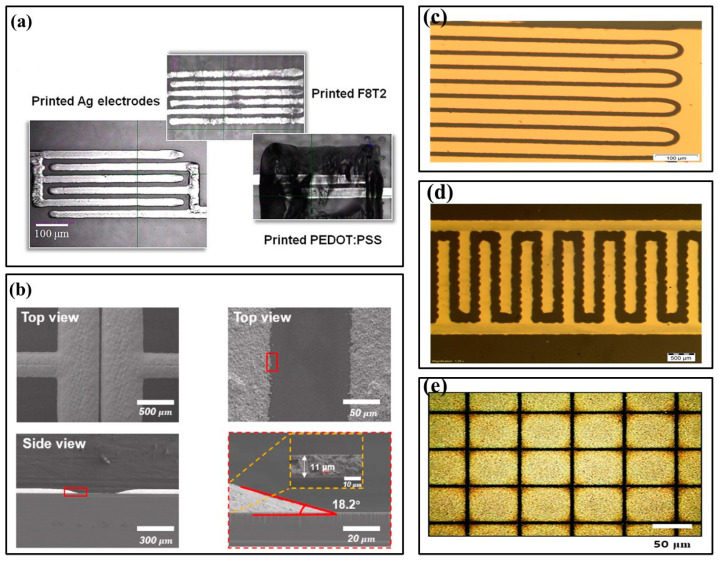
Electrode structure images. (**a**) Optical image of the printed Ag electrodes, F8T2 organic active layer, and poly(3,4-ethylenedioxythiophene):polystyrenesulfonate (PEDOT:PSS) gate electrode [38] (Copyright 2019 Elsevier). (**b**) Screen-printed S/D electrode’s scanning electron microscope (SEM) image [39] (Copyright 2022 Elsevier). (**c**) Optical image of the transistor channel [40] (Copyright 2014 Elsevier). (**d**) Optical microscopy image [41] (Copyright 2015 Elsevier). (**e**) SEM images of carbon nanotube (CNT) electrode patterns [42] (Copyright 2020 Elsevier).

In 2019, Alshammari et al. proposed drop-on-demand (DOD) printing to cure silver nano-particle inks and produce highly conductive silver patterns, which were also sintered with an excimer laser and heat treatment. (Figure 2a) [38]. Sintering conditions optimized for high conductivity produced patterns similar to heat treatment patterns. In this paper, inkjet printing technology was used to print conductive Ag nano-particles (AgNPs), and processed S/D silver electrodes were used to fabricate organic thin film transistors. Screen printing is a popular and straightforward coating technology that uses mesh fabric screens to mask metal interconnects on printed circuit boards. Compared with traditional vacuum deposition technology, screen printing reduces manufacturing costs. In 2022, Wu et al. analyzed the S/D electrodes of organic thin film transistors screen printed with AgNPs ink (Figure 2b) [39]. The optimized AgNPs had high electrical conductivity, excellent pattern fidelity, and good adhesion due to controlled printing conditions, such as printing speed, annealing temperature, and substrate surface modification.

Fabricating thin and smooth electrodes by printing methods is not difficult, especially for those with high resolution and conductivity. However, to achieve better performance, the size and thickness of electrodes must be small. In addition, the overlap degree between the gate and S/D must be minimized. Therefore, electrodes are usually fabricated by photolithography or other non-printing methods. In 2014, Hascinen et al. presented roll-to-roll printing compatible techniques, which used two separately processed substrates laminated together to manufacture organic thin film transistors whose S/D electrodes were patterned by laser ablation (Figure 2c) [40]. In 2015, Vilkman et al. used a high throughput roll-to-roll process to prepare organic top gate transistors with metal electrodes on flexible substrates (Figure 2d) [41]. The transistor consisted of etched S/D Ag electrodes on a polyethylene terephthalate roll, a gravure-printed polymer semiconductor layer, an insulation layer, and a rotary screen-printed Ag gate electrode on top. S/D electrodes were fabricated by patterning roll-to-roll evaporated silver layers with a printable etchant. Each layer was deposited in a continuous rolling process.

In recent years, carbon nano-materials have attracted attention for their high thermal conductivity, excellent mechanical strength, and optional semiconducting properties. Carbon nano-materials have been used in manufacturing semiconductors and electrodes for various electronic devices. They exhibit similar or superior electrical properties to traditional inorganic materials, such as silicon and its oxide semiconductors [42,43]. In 2020, Kwon et al. reported a new method for fabricating composite patterns of submicron-scale carbon nano-tubes and graphene using electrohydrodynamic printing and wettability patterning instead of lithography (Figure 2e) [42]. This technology can be used to fabricate S/D electrodes for OFETs, since S/D electrodes based on carbon nano-materials have the typical electrical properties of p-type OFETs and the hysteresis is minimal.

## 4. Applications of Transistor-Based Biosensors

Table 1 summarizes the modified materials, linear range, and the limit of detection (LoD) of three transistor-based biochemical sensors. The sensors’ detection targets are based on small-molecule biochemical markers in body fluids, which have shown potential for ultrasensitive and wearable biochemical sensors.

### 4.1. Applications of FET Biosensor in Biomarkers

FET is a semiconductor electronic device that uses the electric field effect of controlling the input circuit to adjust the output current. Its basic structure consists of two highly doped n-type regions (i.e., source and drain) on a p-type semiconductor substrate, which covers the insulation layer between the two electrodes to form a gate. The modulation of the current in the semiconductor channel is affected by the combined effect of the gate voltage and electric field generated by the applied voltage between the S/D electrodes.

Traditional FETs use metal oxides as channel layer materials, known as metal-oxide-semiconductor field-effect transistor (MOSFET) sensors, where the biorecognition surface is either an extension of the gate or the gate oxide itself [58]. However, FET performance is easily damaged by aqueous solutions, which may shorten sensor life. To overcome this shortcoming and facilitate label-free detection in body fluids, researchers have proposed extended-gate field-effect transistor (EGFET) sensors that combine ion-sensitive membranes with MOSFET sensors and replace the metal gate with an electrolyte solution and reference electrodes.

**Figure 3:**
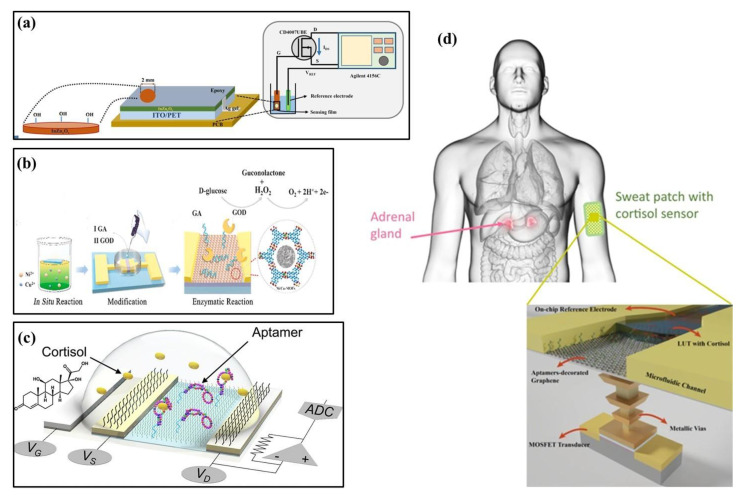
Structures of FET-based biochemical sensors. (**a**) Structure of InZn_x_O_y_ EGFET on PET flexible [26] (Copyright 2020 Elsevier). (**b**) Structural diagram of a field-effect transistor glucose sensor [27] (Copyright 2021 Elsevier). (**c**) Structure of an aptamer-field-effect transistor sensing system [44] (Copyright 2022 Science Advances). (**d**) Structural diagram of a cortisol biosensor. Reproduced from Reference [45] (Copyright 2021 Nature).

In 2020, Palit et al. proposed an indium–zinc oxide (InZn_x_O_y_) sensing membrane on flexible PET using an EGFET biosensor to detect dopamine in organisms (Figure 3a) [26]. To achieve label-free detection of dopamine, InZn_x_O_y_ on PET-based EGFET was functionalized by a synthetic 4-carboxyphenylborate receptor to covalently bind dopamine to the sensing surface. The InZn_x_O_y_ EGFET biosensor has also detected dopamine levels in real samples, such as rat serum and brain. In 2021, Wang et al. reported a glucose sensor based on FETs that used a nickel–copper metal–organic bimetallic framework (Ni/Cu-MOFs) as a channel layer, glutaraldehyde (GA) as a linker, and glucose oxidase (GOD) as a modification layer (Figure 3b) [27]. Due to the synergistic action of Ni^2+^ and Cu^2+^ in MOFs, the sensor had good field effect performance and enzymatic reaction to glucose. In 2022, Wang et al. developed a flexible FET biosensor array, coupling cortisol aptamer to the nano-film In_2_O_3_ FET to detect cortisol (Figure 3c) [44]. The adaptor-FET sensor has universal and modular characteristics, facilitating integration with wearable and mobile devices. Similarly, in 2021, Sheibani et al. proposed a wearable biosensor electronic chip based on a platinum/graphene adapter EGFET for identifying cortisol in biological buffers (Figure 3d) [45]. The sensor achieved high sensitivity, selectivity, and stability without hysteresis using an aptamer as the recognition element. It could also detect nano-molar cortisol levels in a large concentration range.

With the rapid development of nano-material technology in recent years, combining nano-materials and biochemical sensors has received more attention. As a sensing channel for FETs, nano-materials can provide a larger specific surface area, higher carrier mobility, higher transconductance, and lower operating voltage. Its biocompatibility can also immobilize biological probes. Typical nano-materials, such as graphene, carbon nano-tubes (CNTs), MoS_2_, and silicon nano-wires (SiNWs), are commonly used as modification materials for the FET sensing layer [59,60,61,62,63,64].

**Figure 4:**
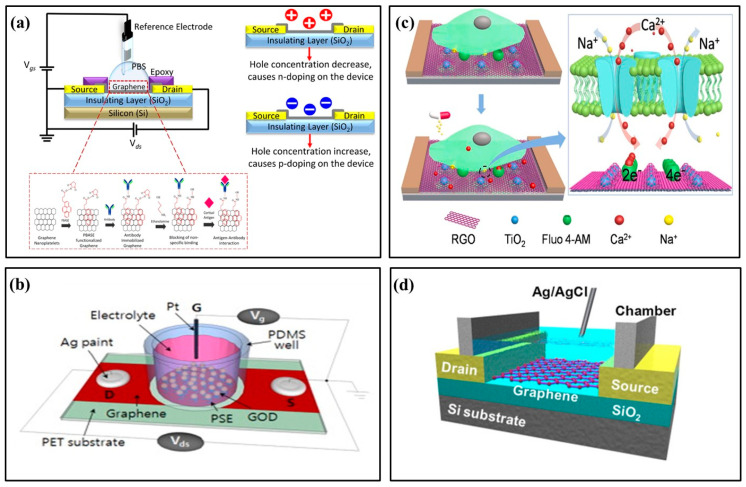
Examples of graphene field-effect transistor biochemical sensors. (**a**) Schematic illustration of the fabricated graphene nano-platelets electrolyte gate field-effect transistor (EGFET) [28] (Copyright 2020 Elsevier). (**b**) Schematic illustration of the solution-gated CVD graphene sensor [46] (Copyright 2022 Elsevier). (**c**) Schematic illustration of the rGON-FET biosensor for cellular Ca^2+^ detection [29] (Copyright 2023 Elsevier). (**d**) Schematic illustration of the selective K^+^ sensor [47] (Copyright 2013 Elsevier).

Graphene FET is widely used in biochemical sensors for its excellent conductivity and high sensitivity to target molecules. Graphene FET biosensors usually deposit two-dimensional graphene material onto the insulation layer between the S/D electrodes. In 2020, Nasyifa et al. proposed fixing monoclonal cortisol antibodies on graphene surfaces to detect cortisol specifically. They developed an integrated graphene nano-plate-based immunosensor based on EGFET (Figure 4a) [28]. Kwak at el. proposed a flexible glucose sensor using graphene FET grown by chemical vapor deposition (CVD) [46]. Graphene and linked molecules were functionalized to immobilize enzymes related to glucose catalytic reactions (Figure 4b). Graphene FET sensors have bipolar transfer characteristics and provide good curve-fitting models and high resolutions even when deformed. They can also be used for continuous real-time monitoring. In 2023, a photocatalysis-induced renewable reduced graphene oxide nano-sheet FET (rGON-FET) biosensor was proposed to monitor calcium ions (Ca^2+^) released by cells in real time [29]. The biosensing channel was assembled layer by layer from rGON and titanium dioxide rGO (TiO_2_-rGO) composites (Figure 4c). At the same time, Fluo 4-am, a molecule specific to Ca^2+^, was immobilized on the channel interface and showed high selectivity for Ca^2+^ in the presence of excess amounts of other metal ions. Maehashi et al. proposed an electrolyte-gated graphene FET and prepared a selective K^+^ sensor by modifying graphene FET with selective K^+^ carrier valinomycin (Figure 4d) [47]. With the specific binding of K^+^ with valinomycin in the ion-selective membrane, positive charges gradually accumulate on the surface of the graphene channel, thus obtaining the linear relationship between the electrical characteristics of FET and the ion concentration.

### 4.2. OFET Biosensor Applications in Biomarkers

OFETs refer to transistors that use organic semiconductors as channel modifiers for FET. They are flexible, light, soluble, and biocompatible. Compared with inorganic materials, OFET has many advantages, such as low manufacturing costs, large coverage area, good flexibility, and easy performance adjustment. It has strong application potential in skin electronics, such as software robots, implanted devices, wearable devices, and so on. Similar to FET, OFET consists of five parts: source, drain, gate, semiconductor layer, and insulation layer. There is also a solid dielectric material between the channel semiconductor and the gate. Organic semiconductor molecules bind through weak interactions, such as the π–π interaction or van der Waals force.

The basic principle of OFET is that when the source electrode is grounded, two potentials are applied to the gate and drain electrodes. This process results in two voltages: the gate-source voltage (V_GS_) and the drain-source voltage (V_DS_). The S/D electrodes contact the semiconductor layer, and the insulation layer separates the semiconductor layer from the gate. Therefore, OFET acts as a parallel-plate capacitor with the semiconductor layer and the gate as bipolar plates. Organic semiconductors have low carrier mass, high resistivity, and high intrinsic resistance when no voltage is applied to the gate. Electrostatic induction occurs when a voltage is applied to the gate. Induced charges are generated near the surface of the semiconductor and insulating layers, forming a conductive channel. Therefore, the OFET current can be tuned according to the electric field. The effective channel is located near the dielectric region of the semiconductor, along which current can flow at a sufficient voltage. That is, the closer the analyte approaches the semiconductor/dielectric interface, the greater the impact on OFET-based sensors’ electrical performance.

**Figure 5:**
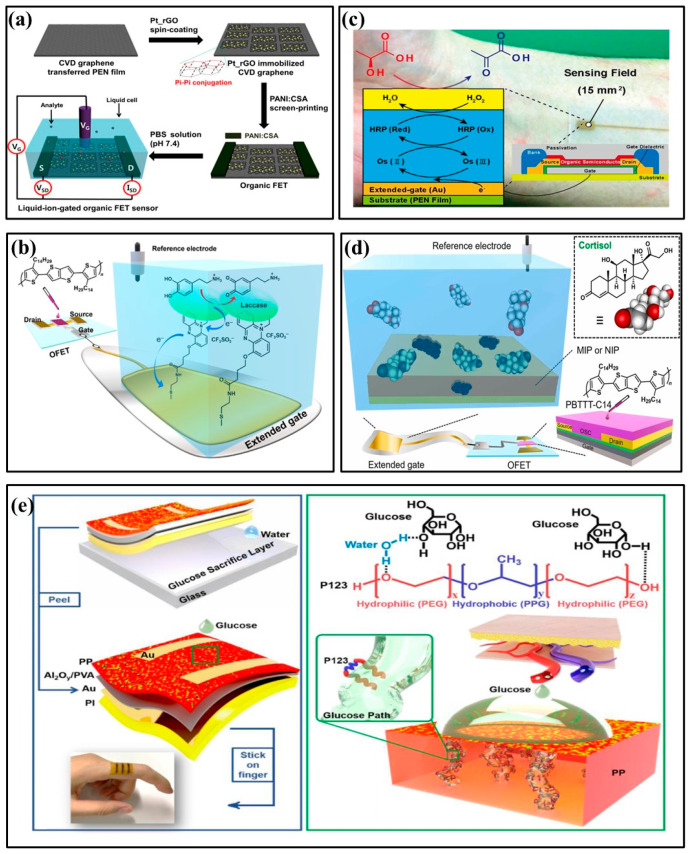
Examples of organic field-effect transistor biochemical sensors. (**a**) Illustrative diagram of the dopamine detection OFET sensor [48] (Copyright 2017 American Chemical Society). (**b**) Schematic illustration of the extended-gate-type OFET-based biosensor for detecting dopamine in human urine [49] (Copyright 2023 Elsevier). (**c**) Schematic illustration of the extended-gate type OFET biosensor for detecting lactate [50] (Copyright 2015 Elsevier). (**d**) Schematic of MIP-OFET structure for detecting cortisol [51] (Copyright 2023 Elsevier). (**e**) Schematic illustration of the flexible glucose OFET sensor [52] (Copyright 2022 Elsevier).

In 2017, Oh J et al. developed an OFET non-enzyme biosensor that used rGO modified by platinum nano-particles (Pt_rGOs) to selectively detect dopamine (Figure 5a) [48]. Pt_rGOs were fixed on a graphene substrate through π–π interaction, which enhanced dopamine oxidation and improved the OFET sensor’s sensitivity to dopamine. In 2023, Ohshiro et al. developed a biosensor based on an extended gate organic field-effect transistor (EGOFET), which was functionalized with laccase and used to detect dopamine in human urine (Figure 5b) [49]. OFET can selectively detect dopamine in solutions containing a variety of interfering substances using an enzyme molecular layer functionalized extended gate electrode. The performance test of the designed OFET device indicated that it could become an effective sensor platform for urine analysis. It could also accurately monitor dopamine in urine. In 2015, Minami et al. proposed an EGOFET for lactic acid detection in aqueous media (Figure 5c) [50]. The OFET’s expanded gate electrode was modified on a flexible plastic film substrate with a layer of lactate oxidase and horseradish peroxidase osmium-redox polymer, enhancing the sensor’s high selectivity and sensitivity. In 2023, Yui Sasaki et al. applied molecularly imprinted polymers (MIP) for salivary cortisol detection [51]. Five monomers (i.e., 1,2-diaminobenzene) were electrochemically deposited as sensitive membranes and selective materials for cortisol detection (Figure 5d). The detection limit of the MIP functionalized OFET for cortisol was 0.72 μg/L, which can be used to detect salivary cortisol quantitatively.

Although OFET has shown promising applications in biochemical sensors, organic materials perform poorly in aqueous environments. Therefore, applying OFET to aqueous solutions needs further exploration. In 2022, Shi et al. devised a groundbreaking approach, and they increased OFET’s affinity for aqueous solutions by adding amphiphilic molecules to hydrophobic semiconductors (Figure 5e) [52]. By providing good wettability between OFETs and aqueous solutions, OFETs can sense aqueous solutions better. This enables selective and specific detection of glucose, raffinose, starch, and lactic acid in aqueous solutions. In particular, this article developed a portable real-time glucose detection system by combining OFET, wireless transmission, and smartphones, which triggers a warning when the glucose concentration exceeds the limit.

### 4.3. Applications of OECT Biosensors in Biomarkers

Wrighton et al. proposed a new electrochemical device, the OECT, in 1984, inspired by the wide application of conjugated materials in OFET [65]. If the organic semiconductor material between the source and drain allows ion infiltration, charge accumulation may occur not only at the channel/electrolyte interface but also at the conjugated polymer. The operating principle of OECT is to apply a gate voltage to the electrolyte and inject ions into the organic semiconductor channel. The drain current reflects changes in the analyte concentration when the oxidation state of the mixed conductor and conductivity of the active layer are altered.

OECTs can directly sense ions and small molecular species, have a low operating voltage, simple structure, are functional in water environments, and are biocompatible. It can convert chemical signals into electrical signals and detect biochemical markers in body fluids. Typical OECT semiconductor polymer channel materials are PEDOT and its derivatives. In recent years, scientists have found that the conductive polymer PEDOT and its complex with PSS have high conductivity, flexibility, and transparency. They have been increasingly applied to wearable and implantable devices, electronic skins, OECTs, and neural interfaces [66]. PEDOT has attracted attention for its mechanical flexibility, ionic conductivity, and facile thin film deposition. It can also be synthesized by solution, gas-phase, or electrochemical polymerization.

**Figure 6:**
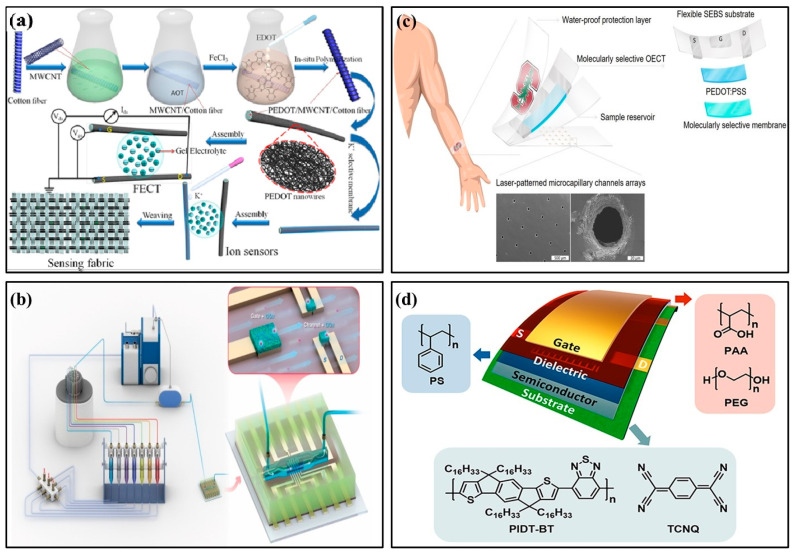
Examples of organic field-effect transistor biochemical sensors. (**a**) Schematic illustration of MWCNT-functionalized PEDOT nano-wires and its applications in K^+^ Sensors based on organic electrochemical transistors [53] (Copyright 2022 Elsevier). (**b**) Schematic of the perfusion system and microfluidic integrated OECT [55] (Copyright 2021 Elsevier). (**c**) Schematic drawings of the patch-type wearable cortisol sensor [56] (Copyright 2018 Science Advances). (**d**) Schematic architecture of flexible OFETs [67] (Copyright 2022 Elsevier).

In 2022, Wang et al. used PEDOT/MWCNT channel functionalized fiber-based electrochemical transistors to detect ultra-low concentrations of potassium ions in the human body (Figure 6a) [53]. Sensors with ion-selective membranes provided good anti-interference ability and were easily fused into fabrics, providing a promising platform for portable health monitoring. In 2018, Mariani et al. developed an OECT sensor for pH monitoring using the PEDOT:BTB:MO composite as the gate electrode. It functioned reliably in double-conversion mode with super-Nernstian sensitivity [54]. In 2021, Koklu et al. proposed an OECT with n-type conjugated polymer channels and a glucose oxidase functionalized gate (Figure 6b) [55]. The device integrated microfluidics with n-type OECT for the first time, monitoring glucose flow in real time. It had higher current and transconductance values and improved signal-to-noise ratio, sensitivity, and LoD. In 2018, Parlak et al. proposed integrating electrochemical transistors with synthetic biomimetic polymer films. Molecular memory layers improve the stability and selectivity of detecting cortisol, a human stress hormone (Figure 6c) [56]. Sensors and laser-patterned microcapillary channel arrays were integrated into a wearable sweat diagnostic platform and applied to skin microfluidic ex situ methods and analysis of real samples. In 2022, Janardhanan et al. studied the effect of functionalized PEDOT nano-structures on the OECT channel layer for cortisol detection in sweat [57]. OECT had a bilayer channel: the upper layer consisted of PEDOT:PSS, and the lower layer was formed by the electrochemical polymerization of monomers EDOT-COOH and EDOT-EG3. The nano-structure-modified OECT device, with its rapid response to cortisol and artificial sweat, has potential clinical utility and practical value in wearable sensors.

### 4.4. Applications of Transistor-Based Sensors in Detecting Electrophysiology and Other Aspects

Flexible transistor-based sensors can detect pressure and electrophysiological signals due to their high transductivity, low operating voltage, mechanical durability, and flexibility. They have broad application prospects in wearable electronics, intelligent sensing, and human motion monitoring. Researchers are also focused on integrating biochemical markers and electrophysiology signals into the same device for simultaneous detection.

In 2017, Nakata et al. developed a wearable, flexible sweat chemical sensor chip for pH measurement, including an ISFET integrated with a flexible temperature sensor [30]. Sweat pH and skin temperature were measured simultaneously in real time through skin contact. This technology has the potential to be developed as a sweat chemical sensor for healthcare and sports. OFETs perform similarly to amorphous silicon devices, with good stretchability and flexibility. These properties have great advantages for wearable temperature and pressure sensors. For example, in 2016, Ren et al. designed a flexible organic temperature sensor array OFET, which can provide two-dimensional temperature information for objects in contact with the human body [31]. During surgery, attaching temperature sensor arrays to the outside of the human body or its organs can provide valuable information on heat distribution for diagnosis and treatment. Polyelectrolyte-gated wearable OFET sensors are limited by severe hysteresis, poor stability, and low sensitivity in practical applications. In 2022, Liu et al. developed a simple media interface passivation strategy to improve the performance of flexible OFETs and polyelectrolyte media. They designed a wearable ultrasensitive pressure sensor (Figure 6d) [67]. A nano-scale polystyrene passivation layer at the polyelectrolyte/semiconductor interface enabled low-voltage polyelectrolyte-gated OFETs with negligible hysteresis and high mobility. Given its stable function, this flexible and low-power OFET pressure sensor can be used as an ultrasensitive wearable pressure sensor to monitor human arm motion.

OECT has become an efficient amplification element for a variety of biosensor scenarios due to its low operating voltage and large cross-conductance. Monitoring ECG signals from human skin is one of the most important non-invasive methods for monitoring human health. Traditional skin ECG signal acquisition methods use passive electrodes to directly transmit the original signal to measurement equipment. However, the signal is easily distorted by noise. In addition to locally amplifying the electrophysiological signal, OECTs suppress noise before the signal is processed by an external amplifier circuit, resulting in significantly improved signal quality. In 2020, Wang et al. proposed a nano-mesh organic electrochemical transistor (NMOECT), a device that could be comfortably attached to human skin and amplify local signals [32]. NMOECT was used to collect and locally amplify an ECG signal truncated at the DC level, achieving a high signal-to-noise ratio of 25.896 dB. This proposed NMOECT could allow for high-quality biological signals from skin electrodes. Furthermore, in 2022, Li et al. designed a flexible OECT for ECG signal acquisition. The sensor exhibited strong resistance to physical distortion and recorded high-quality ECG signals [68]. This flexible and biocompatible OECT could be attached to the right hand via an Ag/AgCl electrode. Another Ag/AgCl electrode was placed on the chest (near the heart). Experimental results show that the signals were distinguishable, and high-quality ECG signals could still be recorded under distortion conditions.

### 4.5. Applications of Transistor-Based Sensors Combined with AI

AI has already been integrated into various scientific fields. ML algorithms have also been used to optimize biomedical sensor design and data analysis. AI algorithms make it possible for POCT to develop into intelligent, precise, automated, and cloud-based iPOCT. Transistor-based biochemical sensors integrated with iPOCT can achieve accurate detection, fast response, and cloud sharing. They will surely play an important role in smart medicine.

In 2019, Min Hsuan Lee proposed a method for predicting OFET charge mobility using machine learning [69]. He optimized the energy levels of the highest occupied molecular orbital and lowest unoccupied molecular orbital of n-type semiconductor materials using gradient enhancement and random forest regression algorithms to model experimental datasets. In 2021, Ma et al. proposed the first MoS_2_ artificial neural network (ANN) chip, which produced hundreds of wafer-level FETs and high-uniformity MoS_2_ thin films. They also implemented a top-gate structure FET using a gate-last process [20]. In 2019, Bian et al. combined FET feature analysis and ML algorithms to distinguish five purine compounds. They used linear discriminant analysis to accurately identify target analytes with multiple characteristics in their sample [70]. In 2018, Rong et al. used support vector machine (SVM) algorithms to analyze small molecular biosensors’ impedance data [71]. They also used this method to detect the interaction between acetone and small chemical sensory proteins and perform equivalent circuit analysis. Moreover, this method can be easily integrated into intelligent mobile devices for quick impedance data detection.

## 5. Conclusions and Prospects

For more than half a century, transistor-based sensors have been widely used in various fields, such as intelligent medical detection and environmental monitoring. Transistor-based biochemical sensors, characterized by high conductivity, low operating voltage, flexibility, and biocompatibility, will surely become a challenging research field.

This review summarizes the applications of three transistor-based biochemical sensors (FET, OFET, and OECT) for detecting biochemical markers, electrophysiology, etc. This review introduces the manufacturing process and preparation methods for transistor electrodes and describes current examples combining sensors and AI. Transistor-based biochemical sensors have been used to detect biochemical molecules because of their high sensitivity, high response speed, and simple operation. With the development of molecular synthesis, surface modification, and device technology, the sensitivity, response speed and selectivity of FET, OFET, and OECT sensors have greatly improved. Using machine learning methods can optimize sensor manufacturing steps and improve data analysis accuracy. Combining biochemical sensors and AI to form an iPOCT system aligns with world technology development trends.

Considering advancements in science, technology, and the medical industry, scientists have focused their research on developing devices that can simultaneously obtain multiple biochemical detection targets and electrophysiological information. The simultaneous detection of multiple target analytes requires an integrated multi-channel biosensor because diagnosing a disease involves monitoring several substances. A biosensor containing an integrated channel for simultaneous detection will greatly save human, material, and financial resources. Against the background of developed technology and multidisciplinary convergence, we believe that transistor-based biochemical sensors will make unprecedented progress in traditional medical detection.

## Figures and Tables

**Table 1:** Three kinds of transistor-based biochemical sensor applications. LoD: limit of detection; MOFs: metal–organic frameworks; rRO: reduced graphene oxide; PBS: phosphate-buffered saline; FBS: fetal bovine serum; PSi-QD: porous silicon quantum dot; CVD: chemical vapor deposition; P123: poly(ethylene glycol)-block-poly(propylene glycol)-block-poly(ethylene glycol) (PEG-PPG-PEG); MIP: molecularly imprinted polymer; PEDOT: poly(3,4-ethylenedioxythiophene); MWCNT: multi-walled carbon nano-tube; BTB: bromothymol blue; MO: methyl orange; MIP: molecularly imprinted polymers; PSS: polystyrenesulfonate.

Types	Materials	Linear Range	LoD	Analyte	Reference
FET	InZn_x_O_y_	1 fM–1 nM	0.523 fM	Dopamine	[26]
	Ni/Cu-MOFs	1 µM–20 nM	0.51 µM	Glucose	[27]
	In_2_O_3_	1 pM–1 µM	1 pM	Cortisol	[44]
	Platinum/Graphene	1 nM–10 µM	0.2 nM	Cortisol	[45]
	Graphene/Monoclonal antibody	1 pg/mL–10 ng/mL	0.85 pg/mL	Cortisol	[28]
	CVD-grown graphene	3.3 mM–10.9 mM	3.3 mM	Glucose	[46]
	TiO_2_-rGO	100 pM–1 mM	100 pM in PBS1 nM in FBS	Ca^2+^	[29]
	Valinomycin	10 nM–1 mM	10 nM	K^+^	[47]
	PSi-QD	1 pM–10 mM	1 pM	H^+^	[11]
OFET	Pt_rGO	100 aM–10 nM	100 aM	Dopamine	[48]
	Laccase	0.029 ppM–0.19 μM	0.029 ppM	Dopamine	[49]
	Lactate oxidase/Horseradish peroxidase	100 nM–1000 nM	66 nM	Lactate	[50]
	MIP/1,2-diaminobenzene	0.72 μg/L–40 μg/L	0.72 μg/L	Cortisol	[51]
	P123	0.5 mg/mL–2 mg/mL	0.5 mg/mL	Glucose	[52]
OECT	PEDOT/MWCNT	1 nM–1000 nM	1 nM	K^+^	[53]
	PEDOT/BTB/MO	0.1 pM–0.1 M	0.1 pM	H^+^	[54]
	N-type polymer/Glucose oxidase	1 nM–1 mM	1 nM	Glucose	[55]
	MIP	0.01 µM–10 µM	0.01 µM	Cortisol	[56]
	PEDOT:PSS/EDOT-COOH/EDOT-EG3	1 fg/m–1 μg/mL	0.0088 fg/mL	Cortisol	[57]

## Data Availability

Not applicable.
